# Detection of *Mycobacterium avium* Subspecies *Paratuberculosis* in Pooled Fecal Samples by Fecal Culture and Real-Time PCR in Relation to Bacterial Density

**DOI:** 10.3390/ani11061605

**Published:** 2021-05-29

**Authors:** Annika Wichert, Esra Einax, Natalie Hahn, Anne Klassen, Karsten Donat

**Affiliations:** 1 Animal Health Service, Thuringian Animal Diseases Fund, Victor-Goerttler-Straße 4, 07745 Jena, Germany; awichert@thtsk.de (A.W.); eeinax@thtsk.de (E.E.); aklassen@thtsk.de (A.K.); 2 Veterinary Office, Johannesstraße 173, 99084 Erfurt, Germany; leaha_2001@web.de

**Keywords:** Johne’s disease, screening, pool size, random sampling, herd level sensitivity

## Abstract

**Simple Summary:**

Paratuberculosis is a worldwide disease causing serious impacts to the dairy industry. Within the context of paratuberculosis control programs, dairy herds have to be classified as either paratuberculosis-positive or paratuberculosis-free with minimum effort but with sufficient reliability. We aimed to estimate the detection rate of positive herds using a combination of random sampling and pooling of five or ten fecal samples. The pooled samples were analyzed with two different laboratory methods (bacterial culture and polymerase chain reaction). Pools of size 10 can be used without significant decrease of detection probability compared with pools of size 5. Analyzing randomly sampled and pooled fecal samples allows the detection of paratuberculosis-positive herds, but the detection probability in herds with only few infected animals (<5.0%) is not high enough to recommend this approach for one-time testing in such herds.

**Abstract:**

Within paratuberculosis control programs *Mycobacterium avium* subsp. *paratuberculosis* (MAP)-infected herds have to be detected with minimum effort but with sufficient reliability. We aimed to evaluate a combination of random sampling (RS) and pooling for the detection of MAP-infected herds, simulating repeated RS in imitated dairy herds (within-herd prevalence 1.0%, 2.0%, 4.3%). Each RS consisted of taking 80 out of 300 pretested fecal samples, and five or ten samples were repeatedly and randomly pooled. All pools containing at least one MAP-positive sample were analyzed by culture and real-time quantitative PCR (qPCR). The pool detection probability was 47.0% or 45.9% for pools of size 5 or 10 applying qPCR and slightly lower using culture. Combining these methods increased the pool detection probability. A positive association between bacterial density in pools and pool detection probability was identified by logistic regression. The herd-level detection probability ranged from 67.3% to 84.8% for pools of size 10 analyzed by both qPCR and culture. Pools of size 10 can be used without significant loss of sensitivity compared with pools of size 5. Analyzing randomly sampled and pooled fecal samples allows the detection of MAP-infected herds, but is not recommended for one-time testing in low prevalence herds.

## 1. Introduction

An infection with *Mycobacterium avium* subsp. *paratuberculosis* (MAP) causes a chronic granulomatous enteritis in cattle, also known as Johne’s disease. After an incubation period of up to 10 years, infected animals develop clinical signs such as intermittent diarrhea, oedema and weight loss despite regular appetite [1,2]. The latter leads to reduced slaughter weights and values [3]. Additional economic losses are attributable to a reduction of milk production [4], increased susceptibility to other diseases, increased culling rates and total losses [5,6].

Not only these economic losses but also the fact that a zoonotic potential of Johne’s disease cannot yet be ruled out yet [7] are reasons for the control of paratuberculosis, particularly as viable MAP can be found in pasteurized consumer’s milk [8,9].

Improvement of animal health or welfare, economic impacts, protection of public health and market access were identified as reasons for control of this widespread disease in a study reviewing paratuberculosis control activities in 48 countries [10]. In more than a half of countries included in that study a control program did not exist, mostly because of lack of economic or animal health resources. Therefore, cost-effective test methods are important for the control of the disease.

The most commonly used tests in control programs are ELISA of serum samples and direct detection of the pathogen in fecal samples by PCR and bacterial culture [10]. Although testing by ELISA is less expensive than fecal culture, ELISA has a lower sensitivity and specificity compared with direct detection of MAP [11,12,13]. The advantage of lower costs of ELISA testing could be undone by necessary follow-up examinations due to the low specificity [14].

A reduction of laboratory costs and required laboratory capacity, compared with testing of all animals within a herd, can be achieved both by taking random samples and pooling of samples [12,15,16]. Furthermore, taking a random sample reduces the workload in taking fecal samples. Therefore, combining both approaches might result in a considerable reduction of testing effort and costs.

In the context of herd-level diagnosis regarding paratuberculosis, several studies have demonstrated that detection probabilities depend on the within-herd prevalence of MAP shedders [17,18,19]. Testing environmental samples from herds with a within-herd prevalence larger than 6% revealed acceptable detection probabilities, but in low prevalence herds the detection probability decreased [19]. Regarding pooled fecal samples in particular, the limits of sensitivity in herds with a very low within-herd prevalence are unknown [20] and the combination of fecal culture and fecal PCR has not been evaluated yet for pooled fecal samples.

The aim of our study was to estimate the detection probability in classifying dairy herds with low prevalence of MAP shedders as MAP-infected or MAP-free applying bacterial culture and real-time quantitative PCR (qPCR), and the combination of both laboratory methods, for detection of MAP and using different pool sizes. We hypothesized that the detection probability at pool level is associated with bacterial density in pools.

## 2. Materials and Methods

Using a set of pretested individual fecal samples from a historically MAP-free herd and samples tested MAP-positive by fecal culture, we simulated a random sampling (RS) in cattle herds. By interspersing different numbers of MAP-positive samples among MAP-negative samples we composed three imitated herds (herd A, B and C) with a size of 300 animals, where an individual sample represented an animal. In herd A, we simulated a within-herd prevalence of 4.33%, i.e., herd A was represented by 13 MAP-positive and 287 MAP-negative individual fecal samples. Herd B and herd C had a within-herd prevalence of 2.0% and 1.0%, respectively (Table A1 in Appendix A).

### 2.1. Fecal Samples

For our study, we used fecal samples from Thuringian dairy herds which had participated in the voluntary Thuringian paratuberculosis control program for several years. Details of the control program are described elsewhere [21]. Briefly, the control program includes annual testing of all cows within a herd by fecal culture. Herds certified as ‘MAP-free’ are retested biennially. The fecal samples were taken rectally using a single-use examination glove and a plastic cup with a barcode of the laboratory for each individual sample. Bacterial culture was applied to each individual sample and afterwards the samples were stored at −20 °C until further processing.

Based on multi-annual test results, we selected individual samples from a MAP-free and a MAP-infected herd for further analysis. In both herds, the cows were of Holstein Friesian breed and housed in free stalls. The MAP-free herd was considered as historically MAP-free, and all individual samples of this herd were MAP-negative in each biennial testing. In the MAP-infected herd 102 MAP shedders were detected by individual culture. Each individual sample was assessed semi-quantitatively using an individual sample score according to the following scheme: − = 0 colonies (score 0); + = 1–10 colonies (score 1); ++ = 11–50 colonies (score 2); +++ = 51–100 colonies (score 3) and ++++ = more than 100 colonies (score 4). Amongst the MAP fecal culture-positive samples, only samples with a cycle threshold (Ct) value of less than 40 were selected.

### 2.2. Random Sampling

According to Kube [22], a sample of *n* = 80 was used which was calculated by FreeCalc [23] to confirm freedom from disease in a population of *N* = 300 and to detect a design prevalence of 5% MAP shedders allowing alpha and beta errors of 5%, respectively. Estimates for sensitivity and specificity of the detection of the infectious agent by fecal culture or qPCR were 70% and 100%.

The RS (80 animals out of 300) was carried out ten times per herd by a random number generator software. The selected individual samples of each RS were assembled for pooling to groups of five by chance. Assembling for pooling was repeated five times, i.e., the 80 samples selected during the RS were grouped five times in five different ways. As each herd was sampled ten times and as each sampling procedure resulted in five different group arrangements, 800 different pools of five individual samples resulted per herd. Pools of size 10 were generated by mixing two pools of size 5 which were dedicated to the same RS. To reflect the bacterial density of all individual samples in a pool taken together, the sum of the individual samples’ scores within a pool was calculated.

### 2.3. Pooling and Testing of Pools by qPCR and Bacterial Culture

Each individual sample was mixed thoroughly using a single-use wooden stick. To obtain pools of size 5, 3 g of each individual sample of a group were mixed with a wooden stick until the sample appeared homogenous. Pools that contained at least one MAP-positive individual sample were analyzed by qPCR and bacterial culture.

For qPCR, we used the commercial kit VetMAX MAP Real-Time PCR Screening Kit (Applied Biosystems LLC, Austin, TX, USA) after DNA extraction with the MagMAX-96 Total Nucleic Acid Isolation Kit (Life Technologies GmbH, Darmstadt, Germany) as previously described by Donat et al. [19]. An internal control was used to detect PCR inhibition. In accordance with the manufacturer’s information, samples with a Ct value less than 37 were considered as MAP-positive. Samples with a Ct value between 37 and 40 were considered as inconclusive and samples with a greater Ct value as negative.

The bacterial culture was conducted according to the method described by the Friedrich-Loeffler-Institut in its official manual of diagnostic procedures [24]. Each pooled sample was inoculated on three tubes of Herrold’s Egg Yolk Medium with Mycobactin. After an incubation time of 4 weeks, the cultures were assessed every 2 weeks. To describe the bacterial density of each pool, a growth index (GI) was determined in the week in which MAP specific colonies became visible for the first time. The GI was calculated based on the colony score (CS) in relation to the week of appearance (WA) according to Köhler et al. (2015) where:

GI = CS × 100 ÷ WA

The CS of the WA were documented according to the following scheme: CS 1 = 1–10 colonies; CS 2 = 11–50 colonies; CS 3 = 51–100 colonies and CS 4 = more than 100 colonies per tube.

The mean of the GI of the three tubes belonging to one pool was used for further analysis.

### 2.4. Statistical Analysis

Statistical analysis was performed using Microsoft Excel, version 2010 [25] and R software, version 4.0.2 [26].

For a combination of qPCR and bacterial culture, the results were rated as follows: if the result of qPCR or the result of the bacterial culture of pooled fecal samples or both were positive, the pool was rated as MAP-positive.

The pool detection probability was calculated by dividing the MAP-positive results of pools by the total number of pools containing at least one MAP-positive individual sample.

At herd-level, a test was considered as MAP-positive if at least one pool taken at a RS showed a positive result. The herd-level detection probability was determined by dividing MAP-positive herd-level tests by the total number of herd-level tests.

In order to evaluate if there was a significant difference between the detection probability of the two different pool sizes, Fisher’s exact test was calculated. McNemar’s test was used to detect a significant difference between the detection probability of the different laboratory methods (qPCR versus bacterial culture, and each laboratory method versus the combination of both laboratory methods).

To analyze the correlation between the number of drawn MAP-positive samples per RS and their average number in pools belonging to the respective RS as well as between the bacterial density in a pool and the Ct value of the same pool, Spearman’s rank correlation coefficient (ρ_s_) was calculated. According to Aly et al. [27], Spearman’s rank correlation coefficient is interpreted as follows: |ρ_s_| > 0.75 = ‘excellent correlation’; 0.40 ≤ |ρ_s_| ≤ 0.75 = ‘fair to good correlation’; |ρ_s_| < 0.4 = ‘poor correlation’.

The influence of the bacterial density in a pool on the binary outcome of qPCR and bacterial culture of pooled fecal samples was analyzed using logistic regression. To check if the model fitted the data, the Nagelkerke’s pseudo R^2^ index was calculated. The model fit was assessed as ‘very good’ if Nagelkerke’s index was greater than 0.5 and as ‘good’ if Nagelkerke’s index was greater than 0.4, but not greater than 0.5.

Statistical significance was assumed if the resulting *p*-value was *p* ≤ 0.05.

## 3. Results

On average, 3.1 true MAP-positive fecal samples were among the 80 randomly selected samples in herd A, 2.0 in herd B and 1.1 in herd C (Table A2 in Appendix A).

Altogether, we analyzed 270 pools of size 10 and 287 pools of size 5 by both qPCR and bacterial culture (Table A3 in Appendix A). In total, ten pools (one pool of herd A, seven pools of herd B and two pools of herd C) and all affected random samplings were excluded from further analysis because of incorrect pooling.

In herd A, ten pools of size 10 and of size 5 contained one individual sample whose bacterial density was described with ++++ whereas, in herd B, 12 pools of size 10 and of size 5 contained such a sample, respectively. In herd C, such samples were added to 19 pools of size 10 and 19 pools of size 5.

### 3.1. Pool Detection Probability

Regardless of which laboratory method was applied, pools of size 5 had a slightly higher detection probability of MAP-positive pools than pools of size 10 (Table 1), with all pools containing at least one MAP-positive sample representing the denominator. Noteworthy, the 95% confidence intervals of the two different pool sizes overlapped. The detection probability increased when using combined diagnostic findings of qPCR and bacterial culture for each pool.

### 3.2. Comparison of Pool Sizes and Analysis Methods

Fisher’s exact test showed no significant difference in the detection probability between the two pool sizes, regardless of which analysis method was used (*p* = 0.799 for qPCR and *p* = 0.865 for bacterial culture). For combined diagnostic findings of qPCR and bacterial culture, this was also the case (*p* = 0.799).

McNemar’s test showed a significant difference for pools of size 5 and 10 (*p* < 0.001 for pools of size 5 and *p* < 0.001 for pools of size 10) when analyzing the pools with bacterial culture only versus evaluating the samples after testing them with qPCR and culture. This was also the case for the comparison of qPCR and combined diagnostic of qPCR and bacterial culture (*p* < 0.001 for pools of size 5 and *p* < 0.001 for pools of size 10). We found no significant difference between bacterial culture and qPCR only (*p* = 0.572 for pools of size 5 and *p* = 0.655 for pools of size 10).

### 3.3. Bacterial Density and Results of qPCR and Bacterial Culture

**Figure 1:**
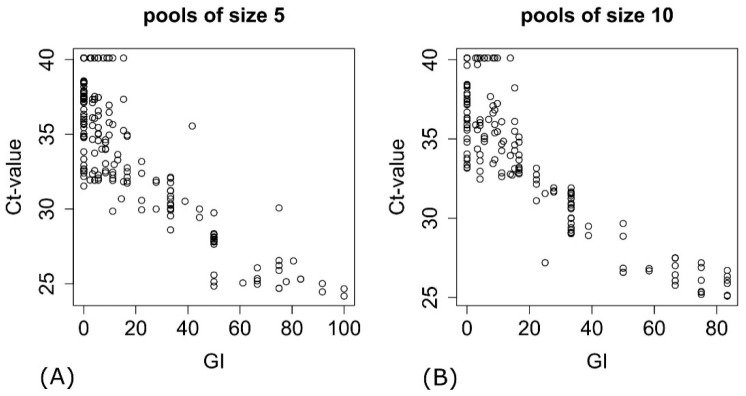
Association between the bacterial density displayed by the growth index (GI) of a sample and the corresponding cycle threshold (Ct) value measured by real-time quantitative PCR. (**A**) Pools of size 5; (**B**) Pools of size 10.

A good correlation was detected between the number of drawn MAP-positive samples and their average number in pools of size 5 (ρ_s_ = 0.69) and 10 (ρ_s_ = 0.75). An excellent correlation between the bacterial density and the Ct value was found for pools of size 5 (ρ_s_ = −0.81) as well as for pools of size 10 (ρ_s_ = −0.83) (Figure 1).

**Figure 2:**
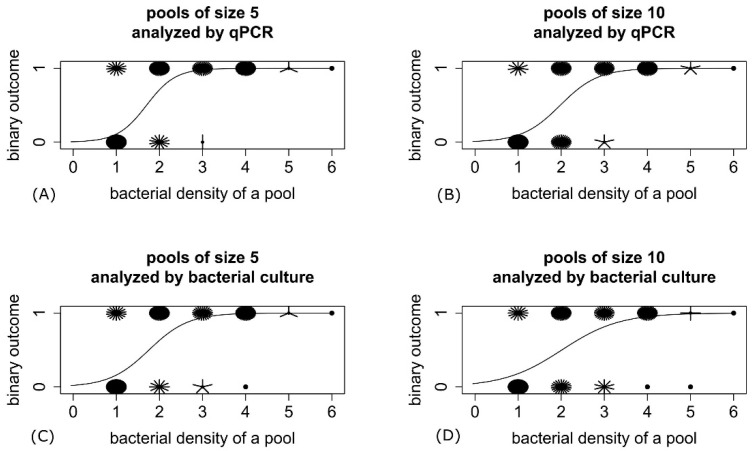
Sunflower plots of the bacterial density in a pool, displayed by the sum of the individual samples’ scores put into a pool, and logistic regression models showing an influence of the bacterial density on the binary outcome of real-time quantitative PCR (qPCR) (**A**,**B**) and bacterial culture (**C**,**D**) of pooled fecal samples for both pool sizes. *Mycobacterium avium* subsp. *avium* (MAP)-positive results were coded 1, MAP-negative results 0.

The logistic regression showed a significant influence of bacterial density in a pool on the binary outcome (MAP-positive or MAP-negative) of qPCR of pooled fecal samples for both pool sizes (*p* < 0.001) (Figure 2). The bacterial density in a pool was also significantly associated with the binary outcome of bacterial culture of pooled fecal samples (*p* < 0.001) (Figure 2). Nagelkerke’s index of the logistic regression models was larger than 0.5, confirming an excellent model fit except for the model regarding pools of size 10 analyzed by bacterial culture, where the model fit was ‘good’ (pseudo R^2^ = 0.47).

### 3.4. Herd-Level Detection Probability

The herd-level detection probabilities, which are shown in Table 1, include five RS of herd B and 15 of herd C that resulted in pooling only feces of MAP-negative samples. In herd A, each RS included at least one MAP-positive sample.

When only one laboratory method was applied, the results of RS of herd A and B showed a slightly higher herd-level detection probability for pools of size 5 than for pools of size 10. In herd C, using pools of size 10 resulted in one more positive herd-level test compared with pools of size 5.

In herd A and B, the highest herd-level detection probability was achieved by means of combined results of qPCR and bacterial culture. This was also the case for herd C when analyzing pools of size 5. In contrast, considering combined results of the two laboratory methods of pools of size 10, the herd-level detection probability of herd C was similar to those achieved by applying qPCR or bacterial culture only.

Pools of size 5 analyzed by both qPCR and bacterial culture showed a higher herd-level detection probability than pools of size 10 in herd A, but in herd B this was the other way round. However, the 95% confidence intervals of the two different pool sizes were overlapping, both in herd A and B. In herd C the detection probability did not differ between the pool sizes when considering combined results of qPCR and bacterial culture.

## 4. Discussion

The objective of this study was to evaluate a diagnostic approach using RS and pooling of five or ten individual fecal samples to classify dairy herds with low prevalence of MAP shedders as MAP-infected or MAP-free. Because the outcome of RS can be estimated by simulation modelling, the influence of the laboratory methods on the pool and herd-level detection probability was the main focus of our study. Our study showed a similar detection probability of bacterial culture and qPCR and demonstrated that using a combination of both increases the pool detection probability.

As demonstrated by logistic regression modelling, the bacterial density and therefore the number of high MAP shedders in a pool is decisive for the pool detection probability. This is consistent with other studies showing that the concentration of bacteria in a pool influences the sensitivity of qPCR [28] as well as of bacterial culture [12,16,29]. One study [20] revealed a linear association between the highest MAP concentration of an individual sample stirred into a pool and the detection probability of that pool. This explains that the highest pool detection probability was achieved in herd C regardless of pool size and laboratory methods despite the low within-herd prevalence in this herd, because the sample of a high shedder in herd C was added to many pools. Our results indicated that the within-herd prevalence determined by counting the number of MAP shedders is not associated with the pool detection probability of qPCR or bacterial culture. This was also observed by Wells et al. [20] in herds with low, moderate or high within-herd prevalence of MAP shedders.

In contrast, considering the herd-level detection probabilities, we estimated the highest detection probabilities in herd B and not in herd C. The low herd-level detection probability in herd C may be due to the fact that RS resulted in 15 completely negative sample sets out of 50 drawings. Our RS resulted in the highest number of MAP-positive samples when they were drawn from herd A in which the most MAP-positive samples were inserted. Thus, we assume that the within-herd prevalence has an influence on the number of MAP shedding animals in a sample set. In our study, the number of drawn MAP-positive samples determined the number of MAP-positive animals in the pools and therefore the bacterial density of a pool. Our results demonstrated that the bacterial density determines the pool level detection probability. Therefore, we suppose that the herd-level detection probability is influenced by the bacterial density in pools and depends on both the number of MAP shedders and the presence of a high shedder in a pool. As previously shown, these drivers are linked and cannot be separated from each other: More high shedding animals can be found in herds with a moderate or high prevalence of MAP shedders (10–30%) than in herds with a lower within-herd prevalence [30].

The detection probability of pooled fecal MAP-positive samples estimated in our study for different pool sizes showed that pooling ten samples instead of only five is not accompanied by significant loss of sensitivity and may be considered to be an effective method. This is in line with the recommendation of other authors who demonstrated that analyzing pools of size 10 is the best strategy with high sensitivity and low costs to identify a herd as MAP-infected or MAP-free by fecal culture [12]. Another study compared different surveillance strategies for paratuberculosis in dairy herds including testing of pooled fecal samples and showed the highest herd-level sensitivity but also high costs for pooled fecal samples, compared with different ELISA-based approaches and environmental sampling [13]. The authors proposed to increase the number of pooled samples from five to ten in order to reduce costs but they assumed that this would lead to reduced sensitivity. In accordance with results of other studies [12,28,31], our results did not confirm this hypothesis. Other studies even found that pooling more samples might lead to a higher sensitivity than pooling fewer samples [28,31]. It is important to mention that the generalization of our results (external validity) and comparability to other studies is difficult because of different sample processing and different applied test methods. All in all, despite different studies providing inconsistent results concerning the pool size with the best sensitivity, our results confirm the concept of pooling ten samples for practical use.

In their paper, Ly et al. [28] suggested that pooling might release components of microorganisms contained in the diet of the animals and that these components could inhibit PCR amplification. As our analyzed fecal samples originated from only two herds, the diet of these herds influenced the detection probability of qPCR. Furthermore, the diet fed could affect the detection probability of bacterial culture as the diet determines the fecal microbiome and therefore the risk of contamination and overgrowth of fecal culture medium [32]. Another factor influencing the detection probability of fecal culture is freezing and thawing of samples. Ly et al. [28] stated that freeze-thawing could also decrease the sensitivity of qPCR as the DNA integrity might be affected.

Nonetheless, our estimated detection probabilities were in the same range as those of other studies [12,16,31] which estimated the sensitivity of bacterial culture of pooled fecal samples. These estimates also seem to be applicable to qPCR because the results of our study showed no significant difference between the pool detection probability of fecal culture and qPCR. Estimates for the sensitivity of qPCR applied on pooled fecal samples from other studies [28,33] support this assumption. In comparison to other studies resulting in detection probabilities of pools of size 5 ranging between 38% and 39% [15] or 34% and 100% [29], our testing approach achieved acceptable detection probabilities.

In herds with a low within-herd prevalence of MAP shedders, on which we focused in our study, a random sample of 80 animals out of 300 contains only few, and in some cases not any, MAP-positive animals. Pooling fecal samples of these animals results in pools with a low bacterial density or even in pools without MAP. As the detection probability is influenced by the bacterial density in a pool which is limited in herds with a low within-herd prevalence, the combination of RS and pooling consequently leads to a limited probability, in our study not higher than 84.78%.

‘Strategic pooling’ was suggested as a possibility to improve the sensitivity of pooled fecal samples [11]. In that study, samples of animals which were in the same age group were pooled in order to have a higher likelihood of putting more than one MAP-positive sample into one pool, resulting in a higher probability of detecting a pool containing MAP. ‘Strategic pooling’ was also considered by other authors [16], who assumed that pooling of animals with a higher likelihood of being MAP-infected could increase the sensitivity of pooled fecal samples. For practical application of this approach, the sampling person in the laboratory staff needs information concerning the age of each animal sampled and has to rank the individual samples by age. This increase in workload makes ‘strategic pooling’ less attractive for practical use.

Other authors [15] suggested repeated testing of herds. Tavornpanich et al. [16] speculated that low shedders would be detected if pooled testing were repeated every 6 to 12 months. Other authors described serial testing of pooled faces as a (cost-)effective method in a paratuberculosis certification scheme [34].

Another possibility to increase the detection probability of MAP-infected herds is to sample more animals within a herd. In consideration of the imperfect sensitivity of the laboratory methods used in our study, a sample size of 80 animals per herd seemed to be a good compromise between cost reduction and achieving high reliable test results. Another study [22] showed that sampling 40 to 80 animals per herd followed by individual fecal culture of the sampled animals resulted in a high detection probability of 15 out of 16 herds (93.8%). The herds tested in this study were also Thuringian dairy herds with a low within-herd prevalence of MAP shedders (<10%) in 13 out of 16 MAP-infected herds and a mean herd size of 253, which is comparable with our study herds. Our results suggest that a larger sample size should be considered if herds suspected to have a very low within-herd prevalence (1–2%) have to be detected. Such a low design prevalence requires a larger number of randomly sampled animals [35]. Increasing the detection probability by multiple testing should be evaluated in further studies.

## 5. Conclusions

We conclude that analyzing pooled fecal samples of randomly sampled animals by bacterial culture can be an effective method of detecting MAP-infected herds. Instead of bacterial culture, qPCR can be used without a loss of sensitivity. Applying a combination of fecal culture and fecal qPCR increases the detection probability but requires more laboratory capacity. Pools of size 10 can be used without significant loss of sensitivity compared to pools of size 5. For pools of size 10 analyzed by both qPCR and culture, the herd-level detection probability ranged from 67.3% to 84.8%. A positive association between bacterial density in pools and pool detection probability was identified. Due to the limited detection probability of a combination of RS and pooling as used in our study, this approach is not adequate to detect herds with a low within-herd prevalence with high reliability and this approach cannot be recommended for one-time testing.

## Figures and Tables

**Table 1:** Pool detection probabilities and herd-level detection probabilities of pools of randomly selected samples (80 out of 300) analyzed by real-time quantitative PCR (qPCR) and bacterial culture.

Detection Method	Pool Size	Detection Probability (%) Herd A	95% CI ^1^ in % Herd A	Detection Probability (%) Herd B	95% CI ^1^ in % Herd B	Detection Probability (%) Herd C	95% CI ^1^ in % Herd C	Detection Probability (%) Overall	95% CI ^1^ in % Overall
Pool detection probability									
qPCR	10	38/131 (29.01)	21.24–36.78	50/88 (56.82)	46.47–67.17	36/51 (70.59)	58.08–83.09	124/270 (45.93)	39.98–51.87
qPCR	5	40/144 (27.78)	20.46–35.09	58/92 (63.04)	53.18–72.91	37/51 (72.55)	60.30–84.80	135/287 (47.04)	41.26–52.81
culture	10	35/131 (26.72)	19.14–34.29	48/88 (54.55)	44.14–64.95	38/51 (74.51)	62.55–86.47	121/270 (44.81)	38.88–50.75
culture	5	41/144 (28.47)	21.10–35.84	52/92 (56.52)	46.39–66.65	38/51 (74.51)	62.55–86.47	131/287 (45.64)	39.88–51.41
Both ^2^	10	47/131 (35.88)	27.66–44.09	60/88 (68.18)	58.45–77.91	38/51 (74.51)	62.55–86.47	145/270 (53.70)	47.76–59.65
Both ^2^	5	54/144 (37.50)	29.59–45.41	65/92 (70.65)	61.35–79.96	39/51 (76.47)	64.83–88.11	158/287 (55.05)	49.30–60.81
Herd-level detection probability									
qPCR	10	29/49 (59.18)	45.42–72.95	35/46 (76.09)	63.76–88.41	34/49 (69.39)	56.48–82.29	-	-
qPCR	5	33/50 (66.00)	52.87–79.13	36/46 (78.26)	66.34–90.18	33/49 (67.35)	54.22–80.48	-	-
culture	10	27/49 (55.10)	41.18–69.03	32/46 (69.57)	56.27–82.86	34/49 (69.39)	56.48–82.29	-	-
culture	5	31/50 (62.00)	48.55–75.45	34/46 (73.91)	61.22–86.60	33/49 (67.35)	54.22–80.48	-	-
Both ^2^	10	33/49 (67.35)	54.22–80.48	39/46 (84.78)	74.40–95.16	34/49 (69.39)	56.48–82.29	-	-
Both ^2^	5	40/50 (80.00)	68.91–91.09	37/46 (80.43)	68.97–91.90	34/49 (69.39)	56.48–82.29	-	-

^1^ 95% confidence interval (CI). ^2^ qPCR and culture.

## Data Availability

Data is contained within the article (Table 1, Table A1, Table A2 and Table A3). Details of laboratory analysis are available on request.

## Appendix A

**Table A1:** Number of individual *Mycobacterium avium* subsp. *paratuberculosis* (MAP)-positive samples classified by semi-quantitative results of bacterial culture used for pooling.

Score	Herd A	Herd B	Herd C
++++	1	1	1
+++	1	1	0
++	2	1	1
+	9	3	1
Sum	13	6	3
Prevalence of MAP shedding animals	4.33%	2.00%	1.00%

**Table A2:** Number of *Mycobacterium avium* subsp. *paratuberculosis* (MAP)-positive samples as drawn by random sampling (RS) of 80 out of 300.

Number of Drawn MAP-Positive Samples	Number of RS in Herd A	Number of RS in Herd B	Number of RS in Herd C
0	0	5	15
1	10	15	20
2	5	10	10
3	15	15	5
4	10	5	0
5	10	0	0
Average	3.1	2.0	1.1

**Table A3:** Number of analyzed pools with one, two or three *Mycobacterium avium* subsp. *paratuberculosis* (MAP)-positive samples per pool.

Number of MAP-Positive Samples Per Pool	Herd	Pools of Size 10	Pools of Size 5
1	A	111	134
	B	80	88
	C	49	49
2	A	17	9
	B	8	4
	C	2	2
3	A	3	1
	B	0	0
	C	0	0
Sum		270	287
